# Extension of Adrenocortical Carcinoma into the Right Atrium

**DOI:** 10.12669/pjms.332.12877

**Published:** 2017

**Authors:** Yulanka Castro-Dominguez, Fatima Samad, Hayder Hashim, Alfonso Waller

**Affiliations:** 1 Yulanka Castro-Dominguez, MD. Department of Cardiology, Yale University School of Medicine, New Haven, CT 06510, USA; 2 Fatima Samad, MD. Aurora Cardiovascular Services, Aurora Sinai/Aurora St. Luke’s Medical Center, University of Wisconsin School of Medicine and Public Health, 2801 W. Kinnickinnic River Parkway, Suite 840, Milwaukee, Wisconsin, 53215 USA; 3 Hayder Hashim, MD. Division of Cardiology, Rutgers – New Jersey Medical School, Newark, NJ 07013, USA; 4 Alfonso Waller, MD. Division of Cardiology, Rutgers – New Jersey Medical School, Newark, NJ 07013, USA

**Keywords:** Adrenocortical Carcinoma, Right Atrium

## Abstract

Adrenocortical carcinoma (ACC) is a rare and highly aggressive malignant neoplasm which can produce intravascular extension into the inferior vena cava (IVC) rarely extend into the right atrium (RA). We report a case of a male patient with large ACC with extension into the IVC and RA. Computed tomography showed a large right adrenal mass with contiguous tumor thrombus extending into IVC and RA with extension to the level of tricuspid valve. Patient underwent combined cardiac and abdominal surgical intervention on cardiopulmonary bypass with removal of the mass.

## INTRODUCTION

Adrenocortical carcinoma (ACC) is a rare and highly aggressive malignant neoplasm with an incidence rate of 1 to 2 cases per million people per year.^1^,^2^ The overall 5-year survival is poor, ranging from 15-44% in reported series.^3^ Multimodality imaging with echocardiogram, computerized tomography (CT), positron emission tomography (PET) and magnetic resonance imaging (MRI) aids not only in establishing the diagnosis but also in anatomic evaluation to determine best surgical approach. We report a case of a large ACC with extension into the inferior vena cava (IVC) and right atrium (RA).

## CASE REPORT

**Fig.1:**
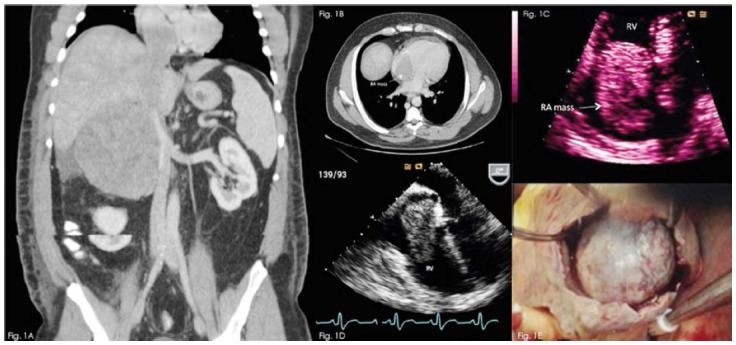
Fig.1A: Computed tomography (coronal view) shows a large, heterogeneous and lobulated mass arising from the right adrenal gland, measuring 10 cm x 12 cm x 22 cm, which extends into the inferior vena cava and right atrium. Fig.1B: Computed tomography (axial view) reveals a filling defect caused by the tumor which occupies the entire right atrium to the level of tricuspid valve. RA = right atrium Fig.1C: Transthoracic echocardiogram (apical 4 chamber zoomed-in view) of the right atrial mass prolapsing through the tricuspid valve into the right ventricle. RA = right atrium; RV = right ventricle. Fig.1D: Transesophageal echocardiogram (mid esophageal 4 chamber view) showing the right atrial mass prolapsing through the tricuspid valve in to the right ventricle. RA = right atrium; RV = right ventricle. Fig.1E: Intraoperative photograph of the right atrial mass.

A 42-year-old male with medical history of hypertension and pre-diabetes presented with a 3-month history of fatigue and intermittent abdominal pain without exacerbating or relieving factors. Physical examination revealed right sided abdominal fullness but no palpable mass. Laboratory studies, including cortisol and urine metanephrine levels, were unremarkable. CT of the abdomen revealed a large right adrenal mass with contiguous tumor thrombus extending into IVC and right atrium RA with extension to the level of tricuspid valve (TV) (Fig.1A and 1B). Transthoracic (Fig. 1C) and transesophageal echocardiogram (Fig. 1D) showed a heterogeneous mass in the RA prolapsing through the TV into the right ventricle. Patient underwent combined cardiac and abdominal surgical intervention on cardiopulmonary bypass with removal of the intracardiac tumor and retroperitoneal mass as well as thrombectomy and dissection of the IVC (Fig.1E). Histopathology showed ACC with neuroendocrine differentiation. The post-operative course was uneventful and he was scheduled to initiate adjuvant treatment with mitotane.

## DISCUSSION

Adrenocortical carcinoma is a rare and highly aggressive malignant neoplasm which can produce tumor thrombus extension into the IVC, rarely invade the RA and exceptionally extend across the tricuspid valve.^3^-^5^ Nearly 60% of these tumors present with a clinical syndrome of hormone excess (i.e., Cushing’s syndrome, virilization). When nonfunctional, ACC may present with the clinical manifestations related to tumor growth (abdominal pain/fullness) or as an incidental radiological finding.^1^ Hormonal evaluation, even in asymptomatic patients, is recommended to determine the secretory activity of the tumor.^6^,^7^

As initial study, CT imaging can distinguish benign from malignant adrenal tumors. The high lipid content in adenomas results in low attenuation in nonenhanced CT. Conversely, carcinomas, having less intracytoplasmic fat, will have higher attenuation and greater Hounsfield units than adenomas. Carcinomas also tend to have irregular shape and be of larger size (>4 cm). On delayed contrast-enhanced CT, adrenal carcinomas (as well as other nonadenomas) have delayed washout of contrast material.^8^

MRI also has some added utility in identifying adrenal carcinomas. On conventional MRI, ACC shows equal intensity as liver on T1-weighted images and increased intensity on T2-weighted images. On gadolinium enhanced images malignant lesions show rapid and marked enhancement and a slower washout pattern. With MRI, involvement of the vena cava and cardiac structures is readily identifiable, hence its utility in surgical planning.^9^

PET scanning with fluorodeoxyglucose (FDG-PET), especially when combined with CT, can be highly valuable in cases where an adrenal mass is very suspicious for malignancy. Adrenal carcinomas uptake FDG avidly, however pheochromocytomas and adrenal metastases do so as well. Alternative PET scan tracers, such as 11C-metomidate [MTO] might further improve specificity, by binding to specific enzymes only present in adrenocortical region.

Precise radiological and echocardiographic evaluation of the extent of the tumor is essential to determine surgical approach. Complete surgical resection is the only curative option for localized disease. In cases of cavo-atrial extension complete resection using cardiopulmonary bypass is recommended.^10^ The prognosis of patients with ACC is generally poor. The overall 5-year survival is poor, ranging from 15-44% in reported series.^3^ Given the scarcity of cases there is no conclusive evidence concerning the impact of IVC and atrial involvement in patients with ACC.

### Authors’ Contribution

**YD:** Data acquisition, collection, manuscript writing and revision of final draft.

**FS:** Data collection, manuscript writing and revision of final draft.

**HH and AW:** Manuscript writing and revision of final draft.

**YD** takes the responsibility and is accountable for all aspects of the work in ensuring that questions related to the accuracy or integrity of any part of the work are appropriately investigated and resolved.
